# Goal-directed fluid therapy using stroke volume variation on length of stay and postoperative gastrointestinal function after major abdominal surgery-a randomized controlled trial

**DOI:** 10.1186/s12871-023-02360-1

**Published:** 2023-12-04

**Authors:** Yanxia Sun, Xuan Liang, Fang Chai, Dongjing Shi, Yue Wang

**Affiliations:** 1 Department of Anesthesiology, Beijing Tongren Hospital, Capital Medical University, Beijing, 100730 China; 2 Department of Anesthesiology, Beijing Hospital, National Center of Gerontology, Institute of Geriatric Medicine, Chinese Academy of Medical Sciences, Beijing, China

**Keywords:** Goal-directed therapy, Fluid therapy, Length of stay, Gastrointestinal disorder, Functional, Intraoperative care

## Abstract

**Background and objective:**

The effectiveness of goal-directed fluid therapy (GDFT) in promoting postoperative recovery remains unclear, the aim of this study was to evaluate the effect of GDFT on length of hospital stay and postoperative recovery of GI function in patients undergoing major abdominal oncologic surgery.

**Methods:**

In this randomized, double- blinded, controlled trial, adult patients scheduled for elective major abdominal surgery with general anesthesia, were randomly divided into the GDFT protocol (group G) or conventional fluid therapy group (group C). Patients in group C underwent conventional fluid therapy based on mean arterial pressure (MAP) and central venous pressure (CVP) whereas those in group G received GDFT protocol associated with the SVV less than 12% and the cardiac index (CI) was controlled at a minimum of 2.5 L/min/m^2^. The primary outcomes were the length of hospital stay and postoperative GI function.

**Results:**

One hundred patients completed the study protocol. The length of hospital stay was significantly shorter in group G compared with group C [9.0 ± 5.8 days versus 12.0 ± 4.6 days, *P* = 0.001]. Postoperative gastrointestinal dysfunction (POGD) occurred in two of 50 patients (4%) in group G and 16 of 50 patients (32%) in the control group (*P* < 0.001). GDFT significantly also shorten time to first flatus by 11 h (*P* = 0.009) and time to first tolerate oral diet by 2 days (*P* < 0.001).

**Conclusions:**

Guided by SVV and CI, the application of GDFT has the potential to expedite postoperative recovery of GI function and reduce hospitalization duration after major abdominal surgery.

**Trial registration:**

This study was registered on www.clinicaltrials.gov on 07/05/2019 with registration number: NCT03940144.

**Supplementary Information:**

The online version contains supplementary material available at 10.1186/s12871-023-02360-1.

## Introduction

Prolonged hospital stay not only delays discharge and results in increased use of medical resources and higher costs, but it also predicts greater risk for readmission and short-term mortality [1]. Postoperative gastrointestinal (GI) dysfunction (POGD) is a leading cause of prolonged hospital stay after major abdominal surgery [2, 3]. Appropriate perioperative fluid management has been reported to improve GI function in patients who undergo major abdominal surgery [4]. However, static indices fail to predict fluid responsiveness in the perioperative period [5].

Goal-directed fluid therapy (GDFT), which monitors dynamic indices to increase oxygen delivery and ensure optimal organ perfusion [6], has been reported to improve the outcome after non-cardiac surgeries [7–10]. Previous studies found that GDFT guided by stroke volume variation (SVV) using FloTrac/Vigileo monitor was associated with a reduced length of hospital stay and a lower incidence of POGD in high-risk patients [7, 11, 12]. The FloTrac/Vigileo is a minimal invasive device assessing flow based hemodynamic parameters by pulse contour analysis based on the radial artery pressure signal [13]. This method gained popularity as it is minimally invasive compared to esophageal doppler or pulmonary artery catheter insertion and provides continuous beat-to-beat data [14]. However, the benefit of this strategy of GDFT in low-to-moderate risk patients remains controversial [15]. Therefore, we performed this single-center, randomized, controlled trial to investigate whether SVV-guided GDFT using FloTrac/Vigileo monitor would improve GI function and shorten the length of hospital stay, compared with a standard conventional fluid therapy in low-to-moderate risk patients undergoing major abdominal surgery.

## Materials and methods

This study was conducted as a single-center, prospective, randomized, partly blinded, controlled trial in a tertiary, university affiliated hospital between May 15, 2019 and January 30, 2021 in accordance with the principles of the 2013 Declaration of Helsinki. All experimental protocols were approved by a named institutional was approved by the Institutional Review Board of Beijing Tong Ren Hospital (TR-IRB no:20170828) and was registered at ClinicalTrials.gov (www.clinicaltrials.gov, NCT03940144) on 07/05/2019. Written informed consent forms were obtained from all included subjects. The reporting of the trial adheres to the Consolidated Standards of Reporting Trials (CONSORT) guidelines [16].

### Study population

Patients undergoing elective major abdominal surgery were recruited. Procedures were considered major if listed for resection of gastrointestinal, gynecologic, and urologic cancer with tumor debulking, staging or reconstruction with a risk for significant surgical blood loss.

Exclusion criteria included co-existing congestive heart failure; chronic lung disease; or renal or hepatic dysfunction (creatinine > 50% or liver enzymes > 50% of normal values), and cardiac arrhythmias. Patients under 18 years, pregnant or lactating woman, patients with esophageal or gastric surgical history, and patients undergoing emergency surgery were also excluded from the study.

Patient characteristics, current diagnosis, and a measure of physiologic and surgical risk (Portsmouth physiologic and operative severity score for the enumeration of mortality and morbidity score: P-POSSUM [17, 18]) were collected.

### Enrollment, randomization, and blinding

Patients were randomized allocated on one-to-one basis to either into a standard conventional fluid therapy group (group C) or GDFT group (group G) using a closed envelope system. A research personnel otherwise not involved in the study prepared and sealed the opaque, consecutively numbered envelopes. Patients were blinded to group allocation. Care providers and investigators in the operating room who supported to fluid strategy administration and recorded intraoperative data could not be blinded due to the presence of the cardiac index trending monitor. The postoperative assessors were blinded to the allocation.

### Perioperative care

All patients in this study were treated with a standard enhanced recovery after surgery (ERAS) program (Supplementary file: Appendix 1). All patients received standard fasting protocol. Solid food was allowed up to 6 h before surgery, and clear fluids up to 2 h before surgery. A liquid diet during the 24 h preceding surgery was prescribed if patients received mechanical bowel preparation.

### Intraoperative management

All patients received basic anesthetic monitoring by five-lead-electrocardiogram, pulse oximetry, blood pressure cuff and bispectral index (BIS), at least one peripheral i.v., a central venous catheter and invasive radial arterial blood pressure monitoring. All patients received bilateral quadratus lumborum muscle block with 0.375% ropivacaine 40 ml 30 min before induction under sedation with midazolam (0.02 mg/kg). In both groups, standard general anesthesia was induced with sufentanyl 0.4–0.5 μg/kg, propofol 1.5-2 mg/kg and cisatracrurium 0.15 mg/kg. After intubation of the trachea, the lungs were ventilated with tidal volume of 8 ml/kg of ideal body weight and positive end expiratory pressure (PEEP) of 4 mmHg, respiratory rate was set to maintain normocapnia using a constant fresh gas flow of 2 L/min. Maintenance of anesthesia was performed with 0.9–1.8% end tidal sevoflurane, remifentanyl and propofol to maintain the BIS value of 40%-60% and cisatracrurium boluses were given as needed.

**Fig. 1:**
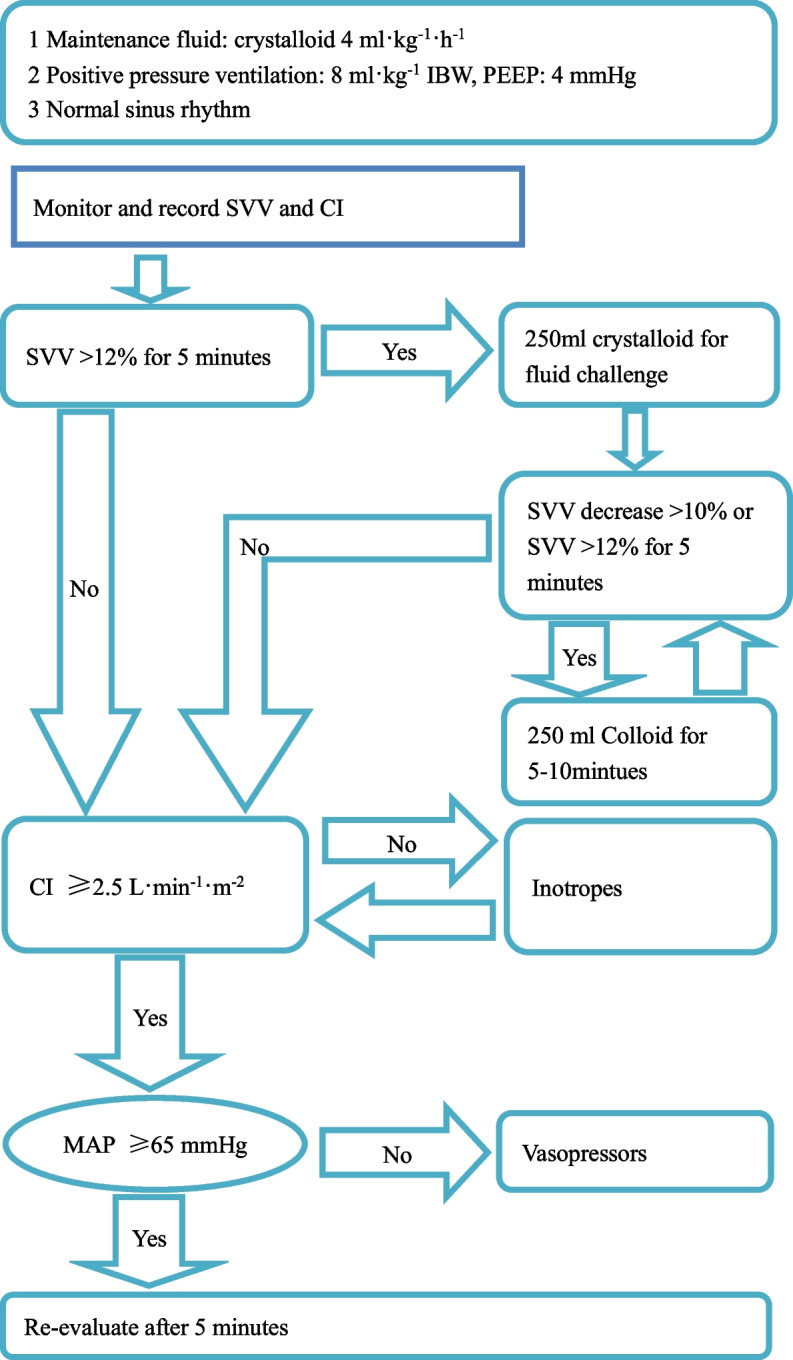
Algorithm for fluid therapy in the operating room in the goal-directed fluid therapy group; CI: cardiac index; MAP: mean arterial pressure; IBW: ideal body weight; PEEP: positive end expiratory pressure; SVV: Stroke volume variation

In group G, the arterial line was connected to the Vigileo monitor (software version 1.14; Edwards Lifesciences, Irvine, CA, USA) via the FloTrac pressure transducer and all intravascular pressure measurements were referenced to mid-axillary line level. The shape of the arterial curve was checked visually for damping throughout the study period. SVV and cardiac index (CI), as indicators for fluid responsiveness during mechanical ventilation and sinus rhythm, were continuously measured. SVV ≤ 12% and CI of at least 2.5L/min/m^2^ were required. 500 mL of crystalloids was infused during induction, followed by a 4 ml/kg/h continuous infusion. If SVV was higher than 12% for over 5 min, a 250 mL bolus of crystalloid was given. Another 250 ml bolus of colloid was administrated if SVV was still higher than 12% or SVV decreased over 10%. If CI value was below 2.5 L/min/m^2^, inotropes were applied to reach this minimum CI, serving as a safety parameter to prevent patients from low cardiac output. If SVV and CI were within the target range but mean arterial pressure (MAP) was below 65 mmHg, vasopressor was started. After the initial assessment, patients were reassessed every 5 min intraoperatively to maintain values according to the study algorithm as illustrated in Fig. 1. Changes in SVV caused by external factors such as pneumoperitoneum, changes in body position, alternation in ventilator settings, or the administration of vasopressor or inotropic drugs did not initiate fluid administration but were noted in the data file.

**Fig. 2:**
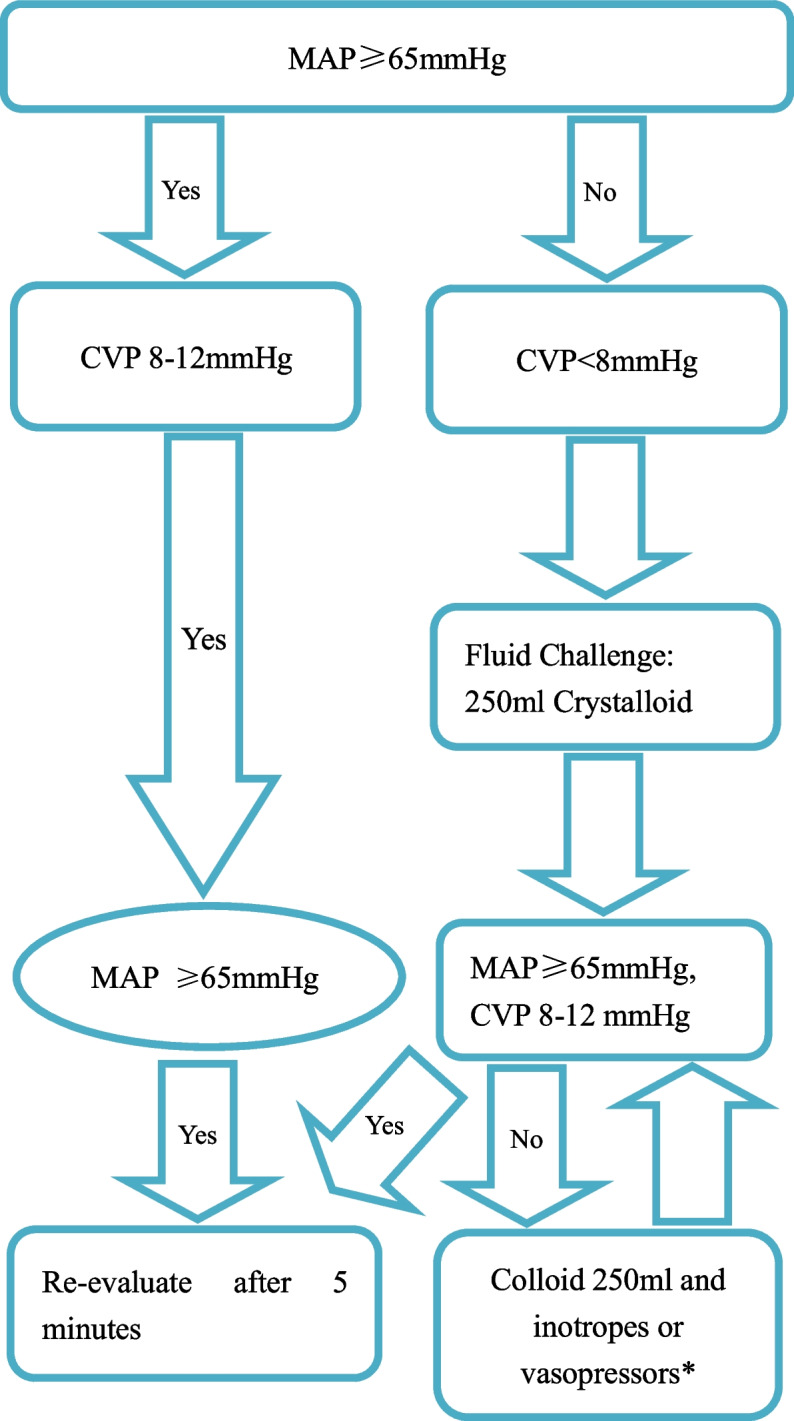
Algorithm for fluid therapy in the operating room in the control group; CVP: central venous pressure; MAP: mean arterial pressure. * The goal did not reach after 500 ml colloid administration, consider inotropes or vasopressors

In group C, MAP was kept between 65 and 90 mmHg, and central venous pressure (CVP) between 8 and 12 mmHg. 500 ml of crystalloids was infused during induction, followed by a continuous infusion of crystalloids (4 ml/kg/h). If the MAP decreased below 65 mmHg, or if the CVP decreased below 8 mmHg, a 250 mL bolus of crystalloids was given. Another 250 ml bolus of colloid was administrated after waiting 5 min if CVP still decreased below 8 mmHg. If the MAP decreased below 65 mmHg and remained unresponsive to fluids, vasopressor or inotrope was given to maintain the MAP above 65 mmHg (Fig. 2).

Normothermia was achieved during surgery with a forced-air warming blanket. Intermittent pneumatic leg compression devices were applied to all patients. No nasogastric tube was given. All patients were given a single intravenous dose of 4 mg of ondansetron as prophylaxis against postoperative nausea and vomiting at the end of surgery. Blood loss was substituted with fluids according to the protocols and a hemoglobin value below 8 mg/dL was considered to be a trigger for transfusion of packed red blood cells. At the end of surgery total catecholamine administration, estimated blood loss, urine output and infused fluids were recorded.

### Postoperative management

Patients in both groups were postoperatively treated with patient-controlled intravenous analgesia (PCIA) with standard analgesic regimens. All patients were transported to the post anesthesia care unit (PACU) unless the intensive care unit (ICU) was indicated because of intraoperative events. The protocol fluid administration continued in the PACU, with all patients in the conventional fluid therapy arm receiving 1 ml/kg/h of balanced crystalloid solution and those in group G receiving 1 ml/kg/h of maintenance and any additional boluses given based on GDFT protocol. All patients were encouraged to early mobilization and early oral nutrition. All patients were follow-up for at least 30 days after surgery by blinded research team members.

### Outcomes

#### Primary outcomes: GI function and length of hospital stay

Length of hospital stay was determined by the period from completion of surgery to discharge. Standardized discharge criteria were used in this study (Supplementary file: Appendix 2) [19]. Postoperative GI function was evaluated daily after surgery by Intake, Feeling nauseated, Emesis, physical Exam, and Duration of symptoms (I-FEED) scoring system (Table 1) [20]. I-FEED score was calculated at 72 h after surgery. The incidence of POGD (defined as I-FEED score ≥ 6), and the incidence of postoperative gastrointestinal intolerance (POGI) (defined as I-FEED score 3–5) were recorded. Time to first tolerate of an oral diet and time to first flatus were also recorded.

**Table 1:** I-FEED scoring system

Scoring item	Intake	Feeling nauseated	Emesis	Exam	Duration of symptoms
Scoring	Tolerating oral diet (0)	None (0)	None (0)	No distention (0)	0–24 h (0)
	Limited Tolerance (1)	Responsive to treatment (1)	≥ episode of low volume (< 100 ml) and non-bilious (1)	Distention without tympany (1)	24–72 h (1)
	Complete intolerance (2)	Resistant to treatment (2)	≥ episode of high volume (> 100 ml) and bilious (2)	Significant distention with tympany (2)	> 72 h (2)

I-FEED, Intake, Feeling Nauseated, Emesis, Exam, and Duration of symptoms. The scoring system attributes 0–2 points for each of the 5 components based on the clinical presentation of the patient and categorizes patients into normal (0–2), postoperative GI intolerance (3–5), and postoperative GI dysfunction (≥ 6)

#### Secondary outcomes

Serum lactate level and blood glucose level were measured at induction and two hours after surgery initiate. Quality of recover score (QoR) based on a previously validated questionnaire (Supplementary file: Appendix 3: QoR-40) [21] was calculated on 1^st^ day, 3^rd^ day, and 5^th^ day after surgery. Time to first postoperative mobilization was recorded. The total number of patients with postoperative complication (except POGD) and the incidence of mortality during study period were recorded from the patient record and by visiting patients on the ward by the investigators. Complications were defined as any deviation from the normal postoperative course, guided by the European Perioperative Clinical Outcome (EPCO) definitions [22].

### Statistical analysis

Our sample size was calculated based on the length of hospital stay using PASS 15.0 software (NCSS, LLC. Kaysville, Utah). According to the findings of our previous meta-analysis [23] and the pilot study of 16 patients, 45 patients needed to be recruited in each group to detect a 3-day mean difference in length of hospital stay between the two groups with standard deviation of 5 in each group, with probabilities of two-sided alpha and beta errors of 0.05 and 0.20, respectively. Sample size was increased to 110 patients to accommodate losses to follow-up and protocol violations. We assumed that a similar sample size would be needed to detect similar difference of postoperative GI function recovery.

All analyses were performed in a modified intention-to- treat population, which included all patients who had undergone both randomization and anesthesia with advanced hemodynamic monitoring for eligible surgery. Continuous data were presented as mean with standard deviation (SD) or median with interquartile ranges (IQR) and binomial data as absolute number (percentage). All data were tested for normality and normal variances and parametric or non-parametric tests were used as appropriate. Fisher’s exact test was used to analyze binomial data. Student t-test or Manne Whitney U-test was used to analyze continuous data as appropriate. Neither multivariable analyses to adjust for preoperative risk nor preplanned subgroup analyses were planned or performed in this study. All tests were two-tailed, and a value of *P* < 0.05 accepted as significant. Statistical analysis was performed using Stata/SE software 16.0 (College Station, TX, USA).

## Results

**Fig. 3:**
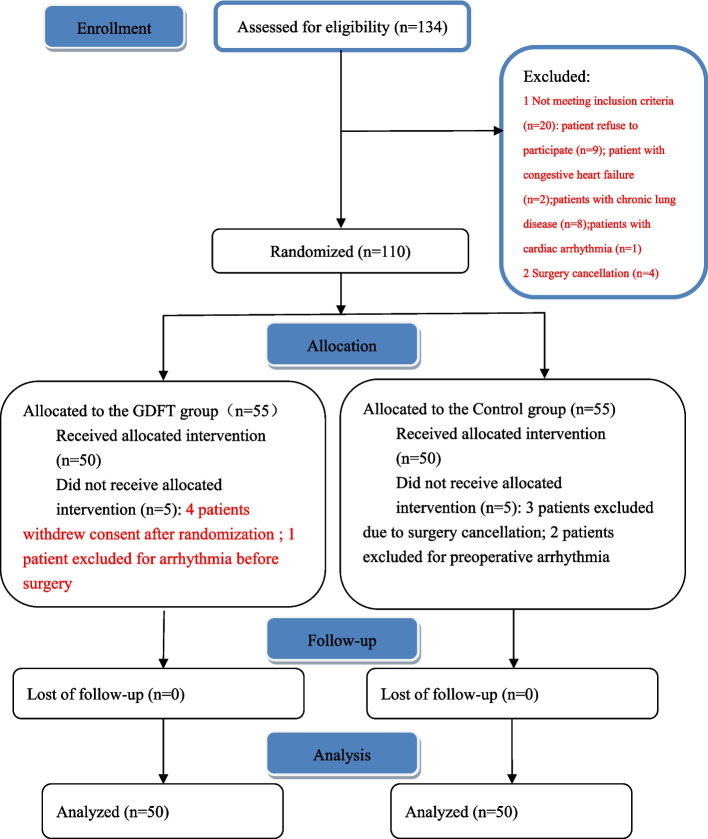
Consolidated Standards of Reporting Trials (CONSORT) diagram

A total of 110 patients were recruited in this study and 10 patients were excluded after randomization based on protocol-defined exclusion criteria: 3 patients excluded due to surgery not proceeding as scheduled, 4 patients withdrew consent after randomization, 3 were found ineligible after consent because of arrythmia on preoperative evaluation. One hundred patients finished this study and data obtained from these patients were used in the analysis (Fig. 3). All evaluable patients were followed for 30 days postoperatively, and none were lost to follow-up.

Comparisons of general characteristics of the study population of both the groups were given in Table 2. No statistical difference was observed in their age, BMI, ASA physical status, sex ratio, duration of anesthesia, duration of surgery, P-POSSUM physical score, P-POSSUM operative score and surgical technique (open or laparoscopy) between the groups.

**Table 2:** Comparison of general characteristics of the study population of both the groups

	Control group (*n* = 50)	GDFT group (*n* = 50)	*P* value
Age, [yr., mean ± SD]	59.2 ± 13.1	64.0 ± 14.0	0.068
Sex ratio, *n* (%)			0.671
Male	34 (68)	32 (64)	
Female	16 (32)	18 (36)	
BMI, mean ± SD	23.8 ± 3.2	23.0 ± 3.4	0.208
ASA physical status, *n* (%)			0.791
1	8 (16)	6 (12)	
2	33 (66)	36 (72)	
3	9 (18)	8 (16)	
Duration of anesthesia, [min, mean ± SD]	250.8 ± 91.0	225.5 ± 87.2	0.195
Duration of surgery, [min, mean ± SD]	212 ± 93.5	191.0 ± 83.5	0.228
P-POSSUM (physiologic score), [mean ± SD]	15.6 ± 2.8	14.9 ± 3.2	0.685
P-POSSUM (operative score), [mean ± SD]	13.4 ± 4.1	14.2 ± 3.8	0.779
Comorbidities, n (%)			
Pulmonary disease	3 (6%)	2 (4%)	
Hypertension	16 (32%)	17 (34%)	
Diabetes	12 (24%)	8 (16%)	
Neurological disease	6 (12%)	8 (16%)	
Coronary artery disease	7 (14%)	9 (18%)	
Type of procedure, *n* (%)			
Gastrectomy	8	7	0.551
Liver resection	3	2	
Whipple	6	4	
Hemicolectomy	20	17	
Rectum resection	5	12	
Radical cystectomy or prostatectomy	3	1	
Radical resection for gynecologic cancer	1	3	
Other oncologic resection procedure	3	3	
Stoma n (%)	7 (14%)	9 (18%)	0.667
Surgical technique, *n* (%)			0.134
Laparoscopy	31 (62)	38 (76)	
Laparotomy	19 (38)	12 (24)	

Data are presented as mean ± SD (standard deviation) or absolute numbers (% percentage)

Data were compared using Student’s t-test, or Fisher’s exact test as appropriate. There were no statistically significant differences between GDFT group and control group

*ASA* American Association of Anesthesiologists, *BMI* Body mass index, *POSSUM* Physiological and Operative Severity Score for the enumeration of Mortality and Morbidity

No specific complications or harm due to the use of the hemodynamics trending monitor or to the application of the study algorithm could be observed. The net amount of fluid administered intraoperatively [1975 ml (1575-2600 ml) in group G versus 2750 ml (2250-3300 ml) in group C, *P* < 0.001] and the amount of crystalloid volume replacement were significantly lower in group G [1600 ml (837 ml-2100 ml) in group G versus 2200 ml (2025-2513 ml) in group C, *P* < 0.001] and more urine output was found in group G [600 ml (300–800 ml) in group G versus 350 ml (200-800 ml) in group C, *P* = 0.041]. The intraoperative fluid balance was also significant lower in group G [1199 ml (800-2750 ml) in group G versus 2116 ml (1100-3100 ml), *p* < 0.001]. No difference was found with regard to administration of vasopressor and inotropes, loss of blood, MAP and heart rate changes, and the amount of colloid administered. Values were given in Table 3 and Table S1 in supplementary file.

**Table 3:** Comparison of the intraoperative fluid management and the use of vasoactive drugs of both group

	Control group	GDFT group	*P* value
Net amount infused, [ml, median (IQR)]	2750 (2250–3300)	1975 (1575–2600)	< 0.001^*^
Crystalloid, [ml, median (IQR)]	2200 (2025–2513)	1600 (837–2100)	< 0.001^*^
Colloid, [ml, median (IQR)]	500 (0–1000)	500 (500–1000)	0.981
Urine output, [ml, median (IQR)]	350 (200–800)	600 (300–800)	0.041^*^
Blood loss, [ml, median (IQR)]	100 (0–350)	100 (50–225)	0.970
Vasopressor or inotrope, *n* (%)	18 (36)	27 (54)	0.072
Norepinephrine, *n*[mg, median (IQR)]	10 [0.04(0.02–0.10)]	13 [0.06(0.04–0.12)]	
Dobutamine, *n*[mg,median (IQR)]	2 [2.62(0–3.50)]	6 [2.55(0–3.50)]	
Phenylephrine, *n*[mg, median (IQR)]	6 [0.05(0.01–0.1)]	7 [0.04(0.02–0.05)]	
Ephedrine, *n*[mg,median (IQR)]	0	1 [6]	

Data are presented as median (IQR, interquartile ranges), or absolute numbers (n, % percentage)

Data were compared using Mann–Whitney U-test, or Fisher’s exact test as appropriate

^*^Statistical significance (*P* < 0.05) compared with control

## Primary outcomes

**Fig. 4:**
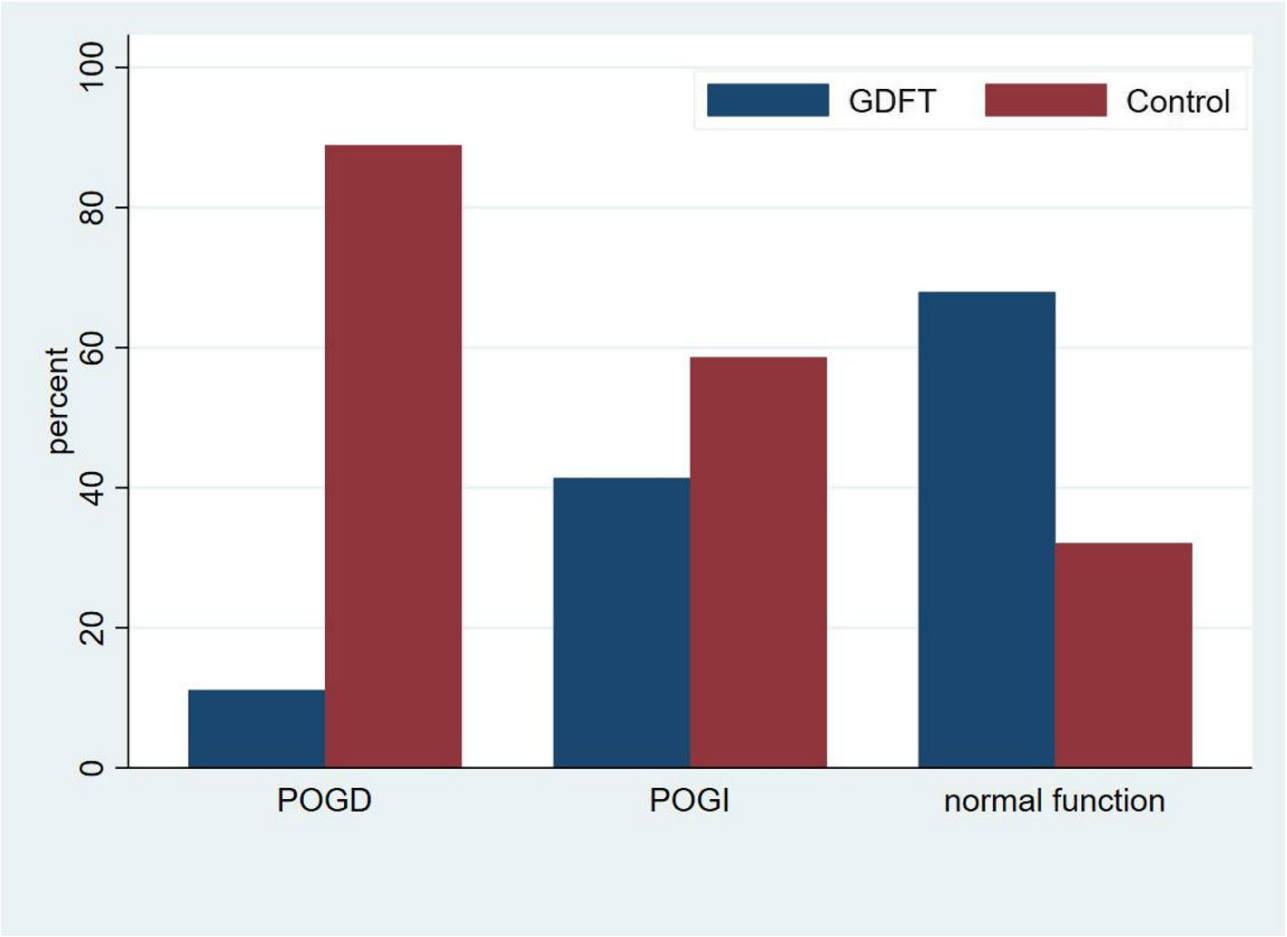
Comparison of gastrointestinal (GI) function using I-FEED score (Intake, Feeling nauseated, Emesis, physical Exam, and Duration of symptoms) after surgery. POGD: postoperative intestinal dysfunction; POGI: Postoperative gastrointestinal intolerance

The mean length of hospital stay was significantly shorter in the group G than that in the group C (9.0 ± 5.8 days versus 12.0 ± 4.6 days, *P* = 0.001) (Table 4). Two of 50 (4%) patients in group G developed POGD, whereas 16 of 50 (32%) patients developed POGD in the group C. Moreover, POGI was observed in 12 patients in group G and 17 patients in group C (*P* < 0.001) (Fig. 4). GDFT significantly shorten time to first flatus by 11 h [28.2 h (9.2–48.0 h) in group G versus 39.4 h (24.9–67.5 h) in group C, *P* = 0.009] and time to first tolerate oral diet by 2 days [4.0 days (2.7–6.0 days) in group G versus 6.0 days (5.0–9.3 days) in group C, *P* < 0.001] (Table 4).

**Table 4:** Comparison of primary outcomes between two groups

	Control group (*n* = 50)	GDFT group (*n* = 50)	*P* value
Time to first flatus, [hours, median (IQR)]	39.4(24.9–67.5)	28.2(9.2–48.0)	0.009^*^
Time to first tolerate oral diet, [days, median (IQR)]	6.0(5.0–9.3)	4.0(2.7–6.0)	< 0.001^*^
The length of hospital stays, [day, mean ± SD]	12.0 ± 4.6	9.0 ± 5.8	0.001^*^

Data are presented as mean ± SD (standard deviation), or median (IQR, interquartile ranges). Data were compared using Mann–Whitney U-test or Student’s t-test as appropriate

^*^Statistical significance (*P* < 0.05) compared with control

## Secondary outcomes

**Fig. 5:**
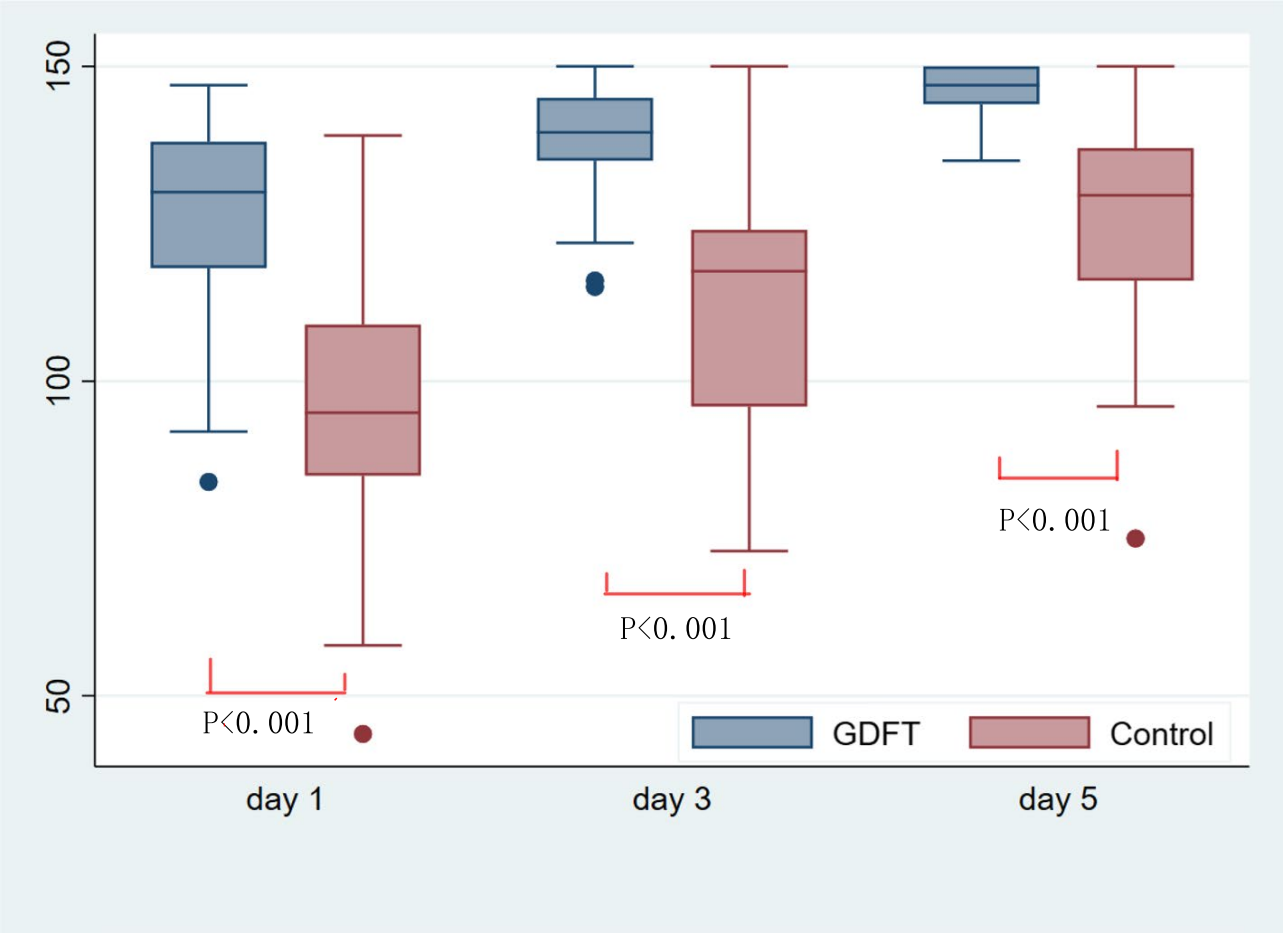
Comparison of quality of recovery (QoR-40) score on day 1, day 3 and day 5 after surgery

Serum lactate level and blood glucose level were not significant different at induction (*P* = 0.652 and* P* = 0.971); however, both of them were higher in group C compared with group G at two hours after initiation of surgery [serum lactate level: 1.4 mmol/L (1.2–1.9 mmol/L) in group C versus 1 mmol/L (0.7–1.2 mmol/L) in group G, *P* < 0.001 and blood glucose level: 7.8 mmol/L (6.1–9.4 mmol/L) in group C versus 6.9 mmol/L (5.7–8.2 mmol/L) in group G, *P* = 0.031] (Table 5). The QoR score was higher in group G on day 1 after surgery [130.0 (118.0–138.0) in group G versus 95.0 (85.0–109.0) in group C, *P* < 0.001], day 3 after surgery [139.5 (135.0–145.0) in group G versus 117.5 (96.0–124.0) in group C, *P* < 0.001], and day 5 after surgery [147.0 (144.0–150.0) in group G versus 129.5 (116.0–137.0) in group C,* P* < 0.001] (Fig. 5). Time to first postoperative mobilization in group G was significantly shorter than that in group C [2.0 days (1.0–3.0 days) in group G versus 2.5 days (1.0–3.8 days) in group C, *P* = 0.025]. The total number of patients with complications other than POGD after surgery were similar between the two groups, except the incidence of postoperative pneumonia. Six patients in group C developed postoperative pneumonia (2 were moderate and the rest were mild), whereas none in group G (*P* = 0.012). The rest complications in this trial were mild, except one patient in the group G died 14 days after laparoscopic gastrectomy secondary to anastomotic rupture and massive hemorrhage (Table 5).

**Table 5:** Comparison of secondary outcomes between two groups

	Control group (*n* = 50)	GDFT group (*n* = 50)	*P* value
Time to first postoperative mobilization, [day, median (IQR)]	2.5 (1.0–3.8)	2.0 (1.0–3.0)	0.025^*^
Serum lactate level [mmol/L, median (IQR)]			
At induction	1.1 (0.9–1.3)	1.1 (0.8–1.6)	0.652
2 h after surgery initiate	1.4 (0.7–1.2)	1.0 (1.2–1.9)	< 0.001^*^
Blood glucose level [mmol/L, median (IQR)]			
At induction	5.9 (5.4–6.9)	5.7 (5.3–7.2)	0.971
2 h after surgery initiate	7.8 (6.1–9.4)	6.9 (5.7–8.2)	0.031^*^
Postoperative complications,* n* (%)	13 (26)	6 (12)	0.072
Arrhythmia	1 (2)	1 (2)	1.000
Pneumonia	6 (12)	0 (0)	0.010^*^
Lower limb venous thrombosis	6 (12)	1 (2)	0.054
Wound infection	5 (10)	2 (4)	0.242
Anastomotic fistula	0 (0)	0 (0)	1.000
Hemorrhage	0 (0)	1 (2)	1.000
30 days mortality,* n* (%)	0 (0)	1 (2)	1.000

Data are presented as median (IQR, interquartile range), or absolute numbers (n, % percentage)

Data were compared using Mann–Whitney U-test, or Fisher’s exact test as appropriate

^*^Statistical significance (*P* < 0.05) compared with control

## Discussion

This study found that using intraoperative GDFT guided by SVV and CI measured by the FloTrac/Vigileo monitor led to improved GI function and a shorter length of stay after major abdominal surgery.

The GDFT group had a mean hospital stay of 9 days, which was 3 days shorter than the control group’s average stay of 12 days. This reduction in postoperative stay is comparable in size to findings from other studies [11, 24–26]. Patients who received GDFT showed a lower incidence of POGD, as well as lower levels of serum lactate and blood glucose during surgery. Additionally, they had a higher recovery score after surgery and were able to mobilize earlier in the postoperative period. One potential explanation for these findings is that GDFT may help to reduce the stress response and tissue edema, while improving systemic perfusion and facilitating GI function recovery. As a result, patients who received GDFT experienced a shorter hospital stay.

The recovery of GI function is a crucial factor in postoperative recovery following major abdominal surgery. Hypovolemia can cause loss of perfusion to the tip of the microvillus, triggering apoptosis and potentially necrosis, which typically requires about 3 days for recovery [27], while excessive fluid administration may inhibit the gastrointestinal transit and result in significant interstitial edema [28]. Previous meta-analyses have indicated that GDFT can reduce the length of hospital stay and aid in the recovery of GI function after major abdominal surgery, particularly in high-risk patients [7, 29–31]. However, the benefits of GDFT in low-to-moderate patients remains controversial. Some studies found that GDFT improved GI function recovery and reduced hospital stay in low-to-moderate risk patients following major abdominal surgery [25, 26, 32]. Others suggest that the treatment benefits may be more modest than previously believed [15, 33–35]. These conflicting findings may be due to differences in the GDFT algorithms used in clinical settings, as there is no clear consensus on the most effective parameter or method of monitoring. In this study, we utilized a GDFT protocol guided by SVV, similar to that used in Benes et al. study [12] and Mayer et al. study [11] which were conducted in high-risk patients. Our findings were consistent with these studies and demonstrated the beneficial effects of GDFT on the length of hospital stay and GI function in low-to-moderate risk patients.

Postoperative GI disorder was commonly referred to as postoperative ileus (POI). However, the heterogeneous definition of POI precludes the ability to ascertain the true incidence of the condition and study it properly within a research setting. In 2018, the American Society for Enhanced Recovery After Surgery (ERAS) and Perioperative Joint Consensus considered forgoing the traditional definition of POI for I-FEED scoring system, a more functional definition of POGD, to precisely describe the clinical manifestations of the GI disorder [20]. POGD was thus used in our study, and the I-FEED score was measured to evaluate GI function. Three categories of postoperative GI function were defined by I-FEED score system, including normal (I-FEED score 0–2), POGI (I-FEED score 3–5) and POGD (I-FEED score ≥ 6). We found GDFT significantly reduced the incidence of POGD (4% in GDFT group versus 32% in the control group) following major abdominal surgery. In addition, tolerance of an enteral diet is one of the fundamental components of postoperative hospital discharge criteria, we found that the time to first tolerate oral diet was shortened by 2 days in the GDFT group compared with the conventional fluid therapy group.

Our study found that SVV-guided GDFT resulted in a significant reduction in total fluid administered (by 29%) and crystalloid administration (by 27%). This suggests that the beneficial effect of GDFT on the recovery of GI function can be attributed to the responsive and guided use of fluids, as well as the avoidance of unnecessary and potentially harmful fluid delivery when hemodynamic goals were met. Additionally, prior research has identified an independent effect of intraoperative volume on postoperative recovery of GI function [36–38]. Overall, these findings highlight the importance of optimized fluid management in improving patient outcomes.

When interpreting these results, it is important to keep the limitations in mind. First, this trial included a mixture of major abdominal oncologic procedures which may potentially influence our results because different types of abdominal surgery led to different mechanical or manipulation forces on the gut and its mesentery. The two groups in this study were comparable with respect to surgical technique, type of procedure and operative POSSUM score. Second, although the data were collected by independent dedicated research personnel not involved in the intraoperative management of patients, we were unable to blind the anesthesiologists and investigators in operating room as to the treatment group, and hence may have introduced bias. However, intraoperative fluid administration in both groups was guided by specific fluid administration protocols, which should minimize bias. Third, the optimal cut-off value for SVV is still uncertain, results of protocols based only on variations itself should be assessed with caution. We used the 12% threshold in our study recommend by Ramsingh [26] for an SVV of over 12% indicates inadequate fluid volume [39]. A dynamic change of CI and MAP were used for decision-making to forestall potential flaws. In addition, we used a fixed tidal volume of 8 ml/kg and excluded patients with irregular heart rhythm to minimize all of potential confounders including vital volume [40] and heart rhythm [41] and assessed a sustained rise of SVV above 12% in a period of five minutes to start an intervention in order to exclude a possible bias due to surgical manipulations or other influences. Fourth, the sample size of this study was calculated based on the endpoint of length of hospital stay. The length of hospital stay is often considered a surrogate endpoint, as it can be influenced by non-medical factors such as healthcare systems and patient preferences. However, in our study, we utilized standardized discharge criteria (detailed in Supplementary File: Appendix 2) that primarily focused on the return of GI function. This allowed us to confidently detect differences in time to the return of GI function. It should be noted that our GDFT strategy was only implemented intraoperatively, and postoperative fluid management was not standardized. Thus, it's possible that poor postoperative fluid management may have negated the benefits of intraoperative fluid optimization.

In summary, our study found that using GDFT guided by SVV and CI with the FloTrac/Vigileo monitor resulted in faster recovery of GI function and shorter hospital stays in low-to-moderate risk patients undergoing major abdominal surgery. However, further large-scale studies are necessary to fully evaluate the impact of this strategy on morbidity and mortality.

### Supplementary Information

**Additional file 1. **

**Additional file 2. **

**Additional file 3. **

**Additional file 4: Table S1.** Intraoperative haemodynamic profile.

## Data Availability

The datasets used and/or analyzed during the current study available from the corresponding author on reasonable request.

## Abbreviations

BIS: Bispectral index
CI: Cardiac index
CVP: Central venous pressure
CONSORT: Consolidated Standards of Reporting Trials
EPCO: European Perioperative Clinical Outcome
GI: Gastrointestinal
GDFT: Goal-directed fluid therapy
ICU: Intensive care unit
I-FEED: Intake, Feeling nauseated, Emesis, physical Exam, and Duration of symptoms
IQR: Interquartile ranges
MAP: Mean arterial pressure
PCIA: Patient-controlled intravenous analgesia
POGD: Postoperative gastrointestinal dysfunction
PEEP: Positive end expiratory pressure
PACU: Post anesthesia care unit
POGI: Postoperative gastrointestinal intolerance
QoR: Quality of recover score
SD: Standard deviation
SVV: Stroke volume variation
