# Supplementary material for: Goal-directed fluid therapy using stroke volume variation on length of stay and postoperative gastrointestinal function after major abdominal surgery-a randomized controlled trial
Source: BMC Anesthesiol. 2023 Dec 4;23:397. doi: 10.1186/s12871-023-02360-1 (PMC10694978; doi:10.1186/s12871-023-02360-1)
Supplement: Supplementary file 1 — Additional file 1. [file 12871_2023_2360_MOESM1_ESM.docx]

Appendix 1

Perioperative enhanced recovery after surgery (ERAS) protocol for major abdominal oncologic surgery in this study

Preoperative management:

1. Pre-operative education. All patients received preoperative assessment and consultation with a surgical specialist and anesthesiologist. Education leaflets on the enhanced recovery procedure, what to expect during and after surgery, analgesia options etc. were made available.

2. Carbohydrate loading beverages 100g the night before and 50g the morning surgery

3. Standard fasting protocol: solid food was allowed up to 6 h before surgery, and clear fluids up to 2 h before surgery. A liquid diet during the 24 h preceding surgery was prescribed if patients received mechanical bowel preparation.

4. Selective use oral mechanical bowel preparation.

5 No premedication

6 All patients received thromboprophylaxis and antibiotic prophylaxis.

Intraoperative management:

1.Anaesthetic management: All patients had general anesthesia; Long acting iv opioids were avoided where possible; All patients received bilateral quadratus lumborum muscle block with 0.375% ropivacaine 40ml 30 minutes before induction under sedation with midazolam (0.01 mg·kg-1).

2. Invasive monitoring and urinary catheterization were considered for all major abdominal oncologic patients.

3. Normothermia was achieved during surgery with a forced-air warming blanket. Intermittent pneumatic leg compression devices were applied to all patients.

4. All patients were given a single intravenous dose of 4 mg of ondansetron as prophylaxis against postoperative nausea and vomiting at the end of surgery

5. Laparoscopic technique was preferred where suitable; Drains were avoided where possible; No nasogastric tube was given.

Post operative care

1. Patients in both groups were postoperatively treated with patient-controlled intravenous analgesia (PCIA) with standard analgesic regimens. All patients were transported to the post anesthesia care unit (PACU) unless the intensive care unit was indicated because of intraoperative events. The protocol fluid administration continued in the PACU, with all patients in the conventional fluid therapy arm receiving 1 ml·kg-1·h-1 of balanced crystalloid solution and those in the GDFT group receiving 1 ml·kg-1·h-1 of maintenance and any additional boluses given based on SV optimization.

2. Regular Paracetamol and NSAID were prescribed unless contra-indicated.

3. Appropriate thromboembolism prophylaxis. Graduated compression stocking were used and prophylactic low molecular weight heparin was given subcutaneously daily at 18:00hrs

4. Care was taken with the prescription of intravenous fluids so as to avoid any excessive daily positive fluid balance. Oral drinks were preferred as the principal route for fluid intake with a recommended daily fluid intake around 2 liters. Urinary catheters were removed on postoperative day 2

5. Early resumption of enteral feeding was promoted, aim to progress from oral fluids to full diet within a few days of the operation.

6. Mobilization

i. Day 0: where practicable, patients were encouraged to sit out of bed, with a target of 2 hours

ii. Day 1: patients were encouraged to have had four 60m walks in the course of the day
