# Supplementary material for: Goal-directed fluid therapy using stroke volume variation on length of stay and postoperative gastrointestinal function after major abdominal surgery-a randomized controlled trial
Source: BMC Anesthesiol. 2023 Dec 4;23:397. doi: 10.1186/s12871-023-02360-1 (PMC10694978; doi:10.1186/s12871-023-02360-1)
Supplement: Supplementary file 2 — Additional file 2. [file 12871_2023_2360_MOESM2_ESM.docx]

Appendix 2

Criteria to determine readiness for hospital discharge following major abdominal surgery:[45]

1. Tolerance of oral intake: Patient should be able to tolerate at least 1 solid meal without nausea, vomiting, bloating, or worsening abdominal pain. Patient should drink liquids actively (ideally 800–1000 mL/day) and not require intravenous fluids infusion to maintain hydration.

2 Recovery of lower gastrointestinal function: Patient should have passed flatus.

3. Adequate pain control with oral analgesia: Patient should be able to rest and mobilize (sit up and walk, unless unable preoperatively) without significant pain (i.e., patient reports pain is controlled or pain score 4 on a scale from 0 to 10) while taking oral analgesics.

4. Ability to mobilize and self-care: Patient should be able to sit up, walk, and perform activities of daily living (e.g., go to the toilet, dress, shower, and climb stairs if needed at home) unless unable preoperatively.

5. Clinical examination and laboratory tests show no evidence of complications or untreated medical problems: Oral temperature should be normal. Pulse, blood pressure, and respiratory rate should be stable and consistent with preoperative levels. Serum hemoglobin concentration should be stable, within acceptable levels. Patient should be able to empty the bladder without difficulty or match preoperative level of bladder function
