# Supplementary material for: Goal-directed fluid therapy using stroke volume variation on length of stay and postoperative gastrointestinal function after major abdominal surgery-a randomized controlled trial
Source: BMC Anesthesiol. 2023 Dec 4;23:397. doi: 10.1186/s12871-023-02360-1 (PMC10694978; doi:10.1186/s12871-023-02360-1)
Supplement: Supplementary file 4 — Additional file 4: Table S1. Intraoperative haemodynamic profile. [file 12871_2023_2360_MOESM4_ESM.doc]

Table S1 Intraoperative haemodynamic profile

|  |  | Control group | GDFT group | P value |
| --- | --- | --- | --- | --- |
| Heart rate (beat.min^-1^) | baseline | 70(55-80) | 71(52-81.5) | 0.491 |
|  | 30 min | 65 (50-73.5) | 68(61-73) | 0.352 |
|  | 60 min | 69 (55-74) | 67(54-80) | 0.320 |
|  | End of surgery | 78 (62-90) | 73(65-82) | 0.214 |
| Mean arterial pressure (mmHg) | baseline | 110(90-125) | 111(85.5-127) | 0.447 |
|  | 30 min | 92(83-110) | 90(81-108) | 0.461 |
|  | 60 min | 85(73-112) | 91(83-99.4) | 0.118 |
|  | End of surgery | 90(73-109) | 92(80-99) | 0.272 |
| Stroke volume Variation (%) | baseline |  | 11 (10-12) |  |
|  | 30 min |  | 10(9-12) |  |
|  | 60 min |  | 11(10-12) |  |
|  | End of surgery |  | 10(8-12) |  |
| Cardiac index (liters.m^-2^) | baseline |  | 2.8(2.3-3.4) |  |
|  | 30 min |  | 3.1(2.7-3.9) |  |
|  | 60 min |  | 2.9(2.5-3.8) |  |
|  | End of surgery |  | 3.0(2.7-3.5) |  |
| CVP (mmHg) | baseline | 10(6-13) | 10(7-12) | 0.997 |
|  | 30 min | 10(8-12) | 11(8-12) | 0.940 |
|  | 60 min | 12(9-13) | 11(8.5-13) | 0.921 |
|  | End of surgery | 11(8-12) | 11(8-12) | 0.994 |
